# May‐Thurner syndrome: A cause of unexplained unilateral leg edema

**DOI:** 10.1002/ccr3.4315

**Published:** 2021-06-24

**Authors:** Masaki Tago, Motoshi Fujiwara, Yoshinori Tokushima, Shun Yamashita, Hidetoshi Aihara

**Affiliations:** ^1^ Department of General Medicine Saga University Hospital Saga Japan

**Keywords:** deep venous thrombosis, leg edema, May‐Thurner syndrome

## Abstract

Physicians should be familiar with May‐Thurner syndrome, characterized by the compression of the left common iliac vein by the right common iliac artery and the vertebral body, resulting in pain and swelling of the left lower extremity and DVT. A 64‐year‐old woman presented with unexplained edema in the left lower extremity. Computed tomography with contrast enhancement revealed that the left common iliac vein was compressed and narrowed by the right common iliac artery and the vertebral body, leading to the diagnosis of May‐Thurner syndrome.

## Graphical abstract

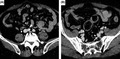

## 1 CASE

**FIGURE 1:**
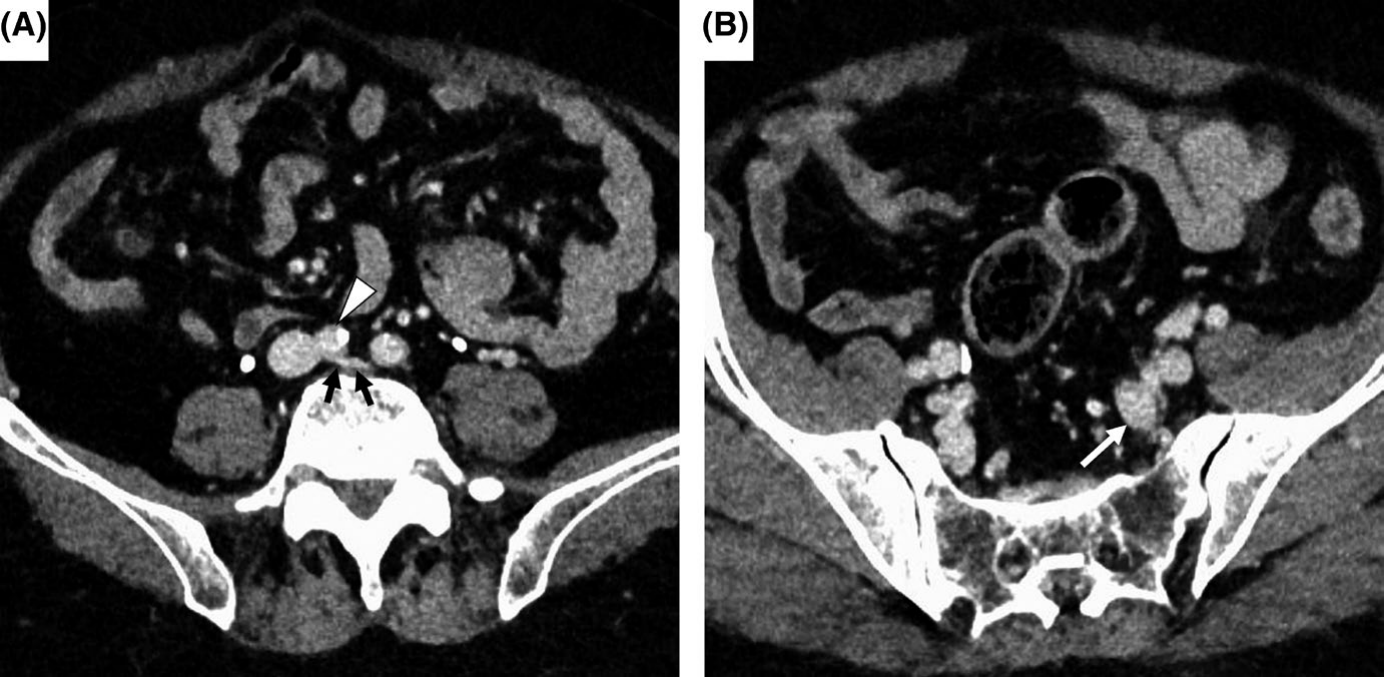
Findings of abdominal computed tomography with contrast enhancement. Computed tomography with contrast enhancement revealed that the left common iliac vein was compressed (A, black arrows) by the right common iliac artery (A, white arrowhead) and the lumbar vertebral body. The distal part of the left internal iliac vein was dilated (B, white arrow)

A 64‐year‐old woman presented with edema in the left lower extremity for 2 months. She was referred to our hospital because a diagnosis could not be made via ultrasonography. Her body mass index was 33 kg/m^2^, and physical examination revealed indurated edema in her left lower leg, without warmth or tenderness. Blood examination revealed no inflammatory reaction, and urinalysis, chest X‐ray, and electrocardiography revealed normal renal function, albumin, thyroid function, D‐dimer, and no abnormalities. Lymphoscintigraphy revealed no lymphatic vessel obstruction. Computed tomography with contrast enhancement revealed that the left common iliac vein was compressed and narrowed by the right common iliac artery and the vertebral body, leading to the diagnosis of May‐Thurner syndrome (MTS) (Figure 1, Video S1).

May‐Thurner syndrome, wherein the left common iliac vein is compressed by the right common iliac artery and the vertebral body, is clinically significant because it causes pain and swelling of the left lower extremity, venous claudication, and deep venous thrombosis (DVT).^1^ Female sex with postpartum, multiparity, oral contraceptives, scoliosis, dehydration, and hypercoagulable disorders is at high risk for MTS.^2^ MTS was found in 14%–32% cases among unselected autopsies, suggesting that there are many undiagnosed cases.^1^ Physicians should be familiar with MTS and correctly diagnose it in cases with edema or DVT in the left lower extremity without any cause or predisposing factors.

## CONFLICT OF INTEREST

None declared.

## AUTHOR CONTRIBUTIONS

MT: involved in literature search, concept, and drafting. MF: involved in literature search, drafting, and clinical care of the patient. YT, SY, HA: involved in conception and revision of the manuscript.

## ETHICAL APPROVAL AND INFORMED CONSENT

The patient gave permission for the publication of this case report. This manuscript conforms to the provisions of the Declaration of Helsinki in 1995 (as revised in Brazil 2013).

## Supporting information

Video S1

## Data Availability

The data that support the findings of this study are available from the corresponding author upon reasonable request.
