# Supplementary material for: May‐Thurner syndrome: A cause of unexplained unilateral leg edema
Source: Clin Case Rep. 2021 Jun 24;9(6):e04315. doi: 10.1002/ccr3.4315 (PMC8223893; doi:10.1002/ccr3.4315)
Supplement: Supplementary file 1 — Video S1 [file CCR3-9-e04315-s001.zip › ccr34315-sup-0002-legends.docx]

**Supporting information**

**Video S1. The video of computed tomography scroll through.**
